# Born Too Soon: Global epidemiology of preterm birth and drivers for change

**DOI:** 10.1186/s12978-025-02033-x

**Published:** 2025-06-23

**Authors:** Ellen Bradley, Hannah Blencowe, Ann-Beth Moller, Yemisrach B. Okwaraji, Francesca Sadler, Anna Gruending, Allisyn C. Moran, Jennifer Requejo, Eric O. Ohuma, Joy E. Lawn

**Affiliations:** 1 Maternal, Adolescent, Reproductive & Child Health (MARCH) Centre, London School of Hygiene & Tropical Medicine, London, UK; 2 School of Public Health and Community Medicine, Institute of Medicine, Sahlgrenska Academy, University of Gothenburg, Gothenburg, Sweden; 3 Partnership for Maternal, Newborn and Child Health (PMNCH), World Health Organization (WHO), Geneva, Switzerland; 4 Department of Maternal, Newborn, Child and Adolescent Health and Ageing, World Health Organization, Geneva, Switzerland; 5 Global Financing Facility for Women, Children, and Adolescents, World Bank, Washington, DC USA; 6 Johns Hopkins University, Baltimore, MD 21025 USA

**Keywords:** Preterm birth, Neonatal, Stillbirths, Data, Risk factors, Prevention, Accountability

## Abstract

**Progress:**

There has been no measurable change in global preterm birth rates in the past decade, in any region. A handful of countries have reduced their preterm birth rates, but only marginally (0.5 percentage points annually), and there has been little progress in availability of preterm birth data globally. An estimated 13.4 million (95% credible interval (CrI): [12.3, 15.2 million]) newborns were preterm or “born too soon” in 2020, 9.9% (95% CrI: [9.1, 11.2%]) of births worldwide. Preterm birth complications remained the top cause of under-5 child mortality globally in 2022, accounting for about 1 million neonatal deaths, similar to figures a decade ago. More encouragingly, some countries have improved data systems to better capture preterm birth information and advancements have been made in gestational age measurement, highlighting targeted efforts towards improving data for action. This paper is part of a series based on the report “Born too soon: decade of action on preterm birth”.

**Programmatic priorities:**

Preventing preterm birth is a critical priority and could be accelerated by focusing on context-specific risk factors, and addressing spontaneous and provider-initiated preterm births, including non-medically indicated caesarean sections. Effective care can prevent 900 000 deaths from complications of preterm birth, particularly among those born before 32 weeks’ gestation.

Stillbirths should be included in data, policies and programmes relating to preterm birth. Most stillbirths occur preterm (an estimated 74.3%) and have a profound, long-lasting impact on families. Addressing stillbirths is essential for reducing the overall burden of preterm birth and minimising loss of human capital.

**Pivots:**

It is important that the data are available and of high quality, plus are used to drive action. We focus on three pivots to improve in the next decade: (1) counting every baby everywhere, including those stillborn, and accurately recording gestational age and birthweight; (2) strengthening national data systems to improve the availability of individual-level data for action, including quality improvement in maternity wards and small and sick newborn care units, plus follow-up for long-term health outcomes including disabilities; and (3) using data to strengthen shared accountability at all levels, from the community to global levels.

**Supplementary Information:**

The online version contains supplementary material available at 10.1186/s12978-025-02033-x.

## Plain language summary

While improved data and increased global focus on newborn survival are positive steps, the number of preterm births remains high at approximately 13.4 million worldwide in 2020. An estimated 1 in 10 babies were born too soon in 2020, and complications related to preterm birth remain the top cause of death in children under 5 based on latest United Nations estimates. Advancing data collection in a unified way (e.g. a core dataset for preterm births) and data use is foundational for change, counting all newborns, both live and stillborn, and with timely and gold-standard assessment of gestational age. Together we can tackle this challenge affecting every family through data-driven decision making, action and transparency at all levels; from local to national to global.

**Supplementary Information:**

The online version contains supplementary material available at 10.1186/s12978-025-02033-x.

## Key findings

• Rates: There has been no change in the preterm rate in the past decade. Globally, 1 in 10 babies is born preterm (<37 weeks’ gestation) (13.4 million (95% credible interval (CrI): [12.3, 15.2 million]) newborns were preterm or “born too soon” in 2020, 9.9% (95% CrI: [9.1, 11.2%])).

• Deaths: In high-income countries, 9 in 10 extremely preterm babies (<28 weeks) survive; whereas fewer than 1 in 10 survive in low-income countries. Preterm birth accounts for 1 million neonatal deaths worldwide, and was the top cause of under-5 mortality in 2022.

• Risk factors: Focusing on known risk factors for preventing preterm birth, and using and improving routine data for the care of preterm babies should be key programmatic priorities.

• Data: Counting every baby, including stillbirths, and tracking the care of vulnerable newborns will ensure that all babies are accounted for in future research, providing data for action and accountability.

**Supplementary Information:**

The online version contains supplementary material available at 10.1186/s12978-025-02033-x.

## Purpose

This paper summarises the latest data and estimates on preterm birth around the world, priorities for prevention based on risk factors, and changes needed to accelerate prevention and improve measurement. Together the seven papers in this supplement were developed from the report “*Born too soon: decade of action on preterm birth”* [1]. The report was part of a campaign to create a movement for preterm birth, linked to the need to accelerate progress for maternal and newborn health and stillbirths, noting slowing of momentum, with flatlining progress for preterm birth being foundational. Content included evidence synthesis of new data, literature reviews and case studies, collated into three themes: (1) *progress* particularly in the last decade; (2) programmatic priorities based on evidence; and (3) pivots needed to accelerate change in the decade ahead. The first paper in this series summarises the definitions and terminology.

## Progress

### Preterm birth trends in rates and numbers, 2010–2020

**Fig. 1:**
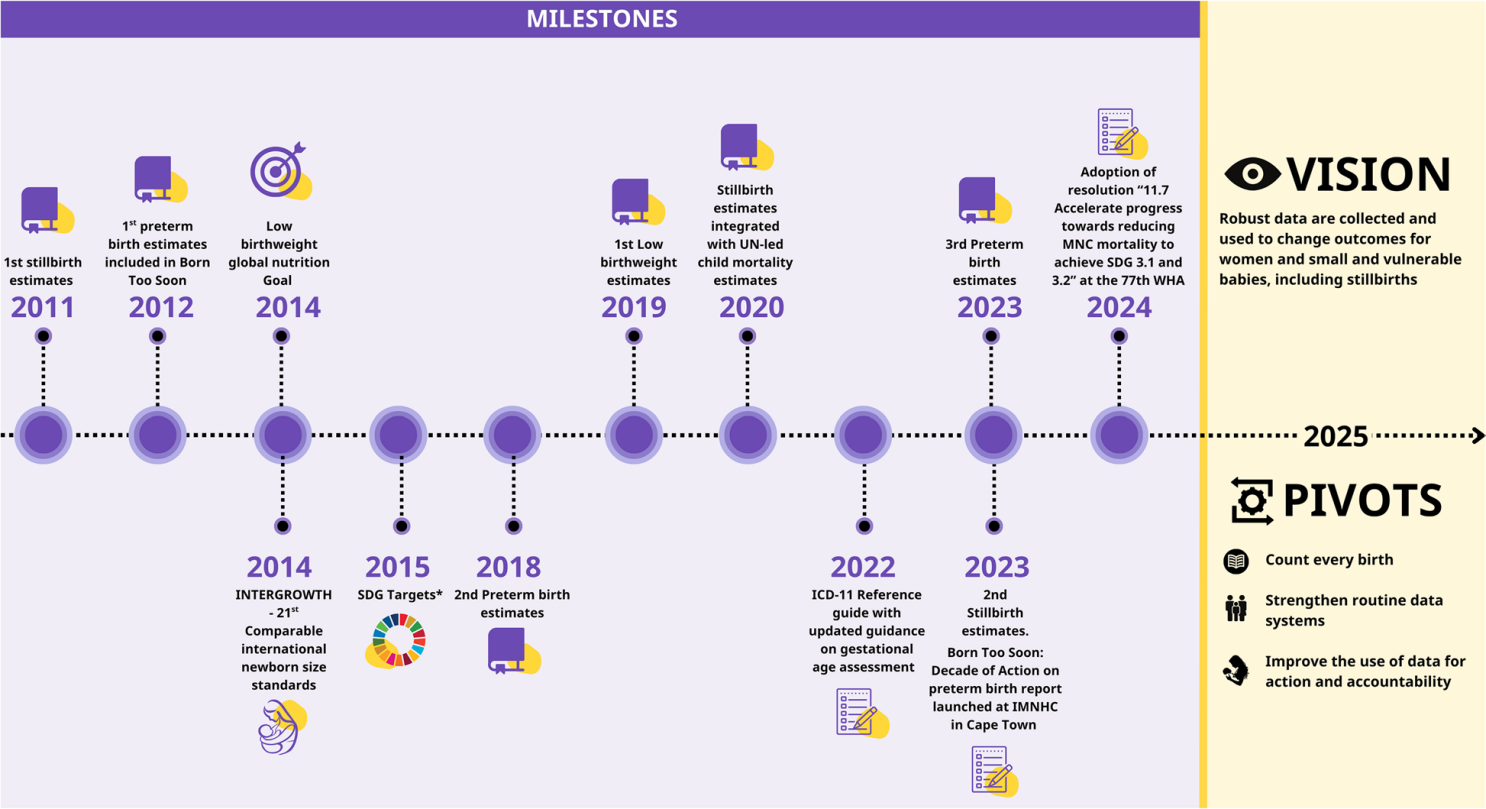
Data and estimates for preterm birth: timeline of progress over the past decade and vision for the next decade SDG – sustainable development goals; ICD – International Classification of Disease [9]. *SDG 3.2 and Every Newborn Action Plan (ENAP) targets to end preventable deaths of newborns and children under 5, with all countries reducing neonatal mortality rate to at least 12 per 1000 livebirths. There is also a linked ENAP stillbirth target reducing stillbirth rate [10, 11]. Preventing preterm birth and improving care for those born too soon is integral to achieving SDG 3.2 and ENAP mortality targets

Preterm birth affects families in every country, on every continent. The past decade has seen mixed progress on collecting and acting on preterm birth data. Policy attention to a healthy start has increased, and the first global goal for neonatal survival was included in the Sustainable Development Goals (SDGs), with a linked target for stillbirths (Fig. 1) [2]. However, preterm birth has not yet directly been included in any high-level policies and measurement frameworks limiting the degree of political focus on reducing preterm birth. While there has been some evidence of an increase in the number of countries collecting data on preterm birth over the last decade, the highest number of countries reporting data is 60 countries. However, early ultrasound dating and innovations in gestational age assessment have improved measurement and some countries have strengthened their routine health information systems to better capture preterm birth rates (e.g. Brazil have improved preterm birth capture over the past 9 years) [3–8]. This highlights targeted efforts towards improving data that can be used for action.

Despite some technological advances, recent estimates by World Health Organization (WHO) and the United Nations Children’s Fund (UNICEF) show no measurable progress in reducing preterm birth rates globally. Preterm birth rates were 9.9% (95% credible interval (CrI) [9.1, 11.2%]) in 2020, compared to 9.8% (95% CrI [9.0–11.0%]) in 2010 [12].There was also no measurable change in preterm birth rates in the highest-burden regions (Southern Asia: 13.3% (95% CrI [10.8–16.5%]) in 2010 and 13.2% (95% CrI [10.8–16.6%] in 2020, and sub-Saharan Africa: 10.1% in both 2010 and 2020 (95% CrI: [8.5–12.7%] in 2010, [8.6–12.7%] in 2020)) [12]. National-level preterm birth rates also changed little between 2010 (5.8%–16.5%) and 2020 (4.1%–16.2%).

Among 76 countries with robust time series data, 10 countries which reduced their preterm birth rates fastest were: Czechia, Austria, Brunei Darussalam, Singapore, Spain, Netherlands (Kingdom of the), Denmark, Hungary, Brazil and Sweden. All these countries reduced their preterm birth rates by more than 5 percentage points between 2010 and 2020, but it is important to note that this equates to an annual average reduction of only about 0.5 percentage points per year. In 14 countries (Russian Federation, Poland, Iceland, Croatia, United Kingdom of Great Britain and Northern Ireland, Bulgaria, Armenia, Bahrain, Ireland, Chile, Georgia, Colombia, the Republic of Korea and North Macedonia) the preterm birth rates increased by more than 5 percentage points in this period, although some of these increases may relate to improved data quality. In 52 other countries the preterm birth rate showed no measurable change (absolute percentage increase <1 percentage points).

**Fig. 2:**
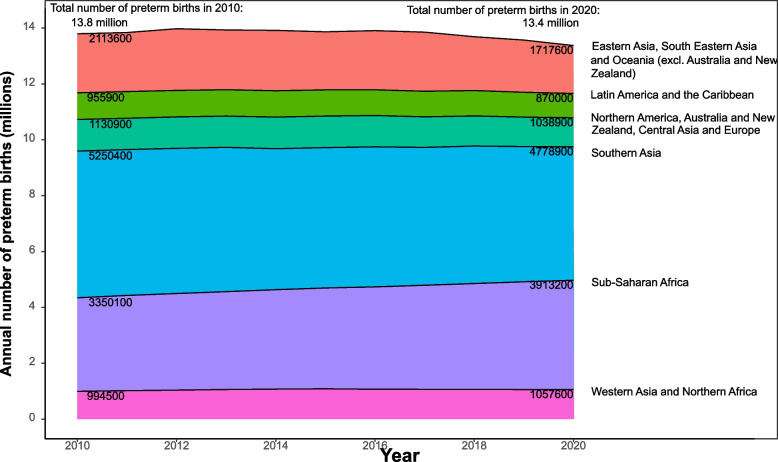
Trends in annual number of preterm births by sustainable development goal regions, 2010–2020 Data from WHO/UNICEF preterm birth estimates, Ohuma, Moller, Bradley et al. [12]. Source: Lawn et al. [13]

The absolute number of babies born preterm decreased slightly from 13.8 million (95% CrI: [12.7–15.5 million]) in 2010 to 13.4 million (95% CrI: [12.3–15.2 million]) in 2020, primarily due to fewer births globally across many regions (Fig. 2) [12]. However, in sub-Saharan Africa the number of babies born preterm increased, with 563 000 more babies born preterm in 2020 than in 2010 (3.4 million (95% CrI: [2.8, 4.2 million]) in 2010, and 3.9 million (95% CrI: [3.3, 4.9 million]) in 2020. This relates to increases in the birth cohort in sub-Saharan Africa, as well as to the lack of reduction in preterm birth rates [12].

Reported effects of the COVID-19 pandemic on preterm birth rates varied across contexts [14–16]. Whilst maternal COVID-19 infection may directly lead to preterm birth including through placental effects, direct impact of maternal systemic illness or vertical transmission, the pandemic was also associated with lower risk of other infections and reduced maternal stress potentially lowering the risk of preterm birth. [17] Obstetric care shifts may increase or reduce preterm birth rates. In published data, preterm birth rates were typically static or slightly increased. Some studies found this was associated with an increased risk of stillbirth, possibly attributed to reduced access to obstetric monitoring and interventions for fetal compromise. However, a meta-analysis of 32 studies and subgroup analysis of four studies found no significant impact on stillbirth rates (adjusted odds ratio (AOR) 1.08, 95% confidence interval (CI) [0.95, 1.23]) and AOR 1.06, 95% CI [0.81–1.38] respectively) [15].

### Preterm rates and numbers in 2020

In 2020, an estimated 13.4 million (95% CrI: [12.3, 15.2 million]) live births were preterm, with 1 in 10 babies “born too soon” (9.9% of all live births (95% CrI: [9.1, 11.2%])). Preterm birth rates vary between regions, the highest occurring in Southern Asia, where 13.2% of babies were born preterm in 2020, compared to fewer than 8% of births in Eastern Asia, South- Eastern Asia, Northern America, Europe, Australia and New Zealand. However, sizeable national variations occur within regions. In Latin America, for example, preterm birth rates estimates for countries with available input data ranged from 5.8% (95% CrI: [5.4, 6.2%]) in Nicaragua to 12.8% (95% CrI: [8.0, 20.4%]) in Suriname [12].

**Fig. 3:**
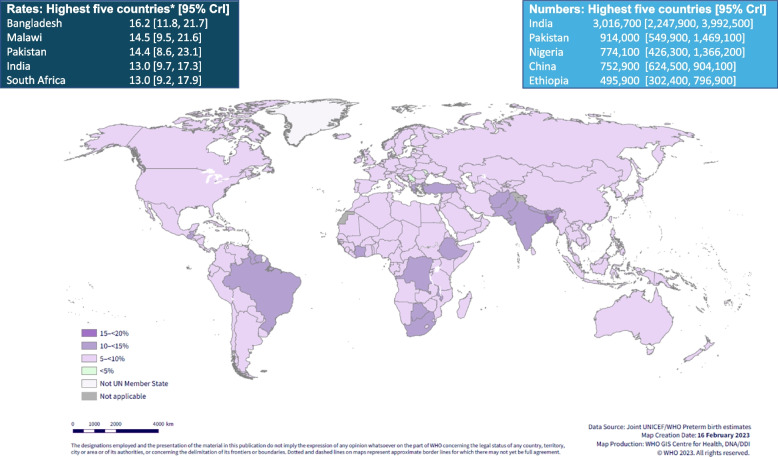
Estimated national preterm birth rates and numbers in 2020 * Only national rates shown. CrI – Credible interval. Source: Adapted from WHO/UNICEF preterm estimates. Ohuma, Moller, Bradley et al. [12]

There is also variation at the national level. According to global estimates in 2020, Bangladesh has the highest preterm birth rate (16.2%, 95% CrI: [11.8, 21.7%]), followed by Malawi (14.5%, 95% CrI: [9.5, 21.6%]) and Pakistan (14.4%, 95% CrI: [8.6, 23.1%]). Although the highest rates are predominantly in low- and middle-income contexts (Fig. 3), rates of 10% or higher persist in some high-income countries, such as Greece (11.6%, 95% CrI: [10.9, 12.3%]) and the United States of America (10.0%, 95% CrI: [9.6, 10.4%]). The nine countries that had the highest preterm rates in 2010 were the same in 2020, and in the same order. The countries with the lowest preterm rates were Serbia (4.1%, 95% CrI: [3.8, 4.4%]) and the Republic of Moldova (5.0%, 95% CrI: [4.0, 6.1%]) (Table 1) [12].

**Table 1:** Countries with preterm birth rate of 10% or higher in 2010 and 2020

**2010**			**2020**		
	**Country**	**Preterm birth rate per 100 live births** (95% Crl^a^)		**Country**	**Preterm birth rate per 100 live births** (95% Crl^a^)
1	Bangladesh	16.4 (12.1–21.8)	1	Bangladesh	16.2 (11.8–21.7)
2	Malawi	14.6 (9.7–21.6)	2	Malawi	14.5 (9.5–21.6)
3	Pakistan	14.3 (8.6–22.9)	3	Pakistan	14.4 (8.6–23.1)
4	Occupied Palestinian territory, including east Jerusalem	13.2 (7.0–23.6)	4	Occupied Palestinian territory, including east Jerusalem	13.2 (7.1–23.4)
5	India	13.1 (9.9–17.2)	5	India	13.0 (9.7–17.3)
6	South Africa	12.9 (9.3–17.7)	6	South Africa	13.0 (9.2–17.9)
7	Ethiopia	12.9 (7.8–20.9)	7	Ethiopia	12.9 (7.9–20.7)
8	Suriname	12.8 (7.8–20.5)	8	Suriname	12.8 (8.0–20.4)
9	Democratic Republic of the Congo	12.4 (7.7–19.4)	9	Democratic Republic of the Congo	12.4 (7.8–19.5)
10	Brazil	12.0 (11.4–12.6)	10	Côte d’Ivoire	11.7 (6.2–21.0)
11	Côte d’Ivoire	11.7 (6.3–21.3)	11	Greece	11.6 (10.9–12.3)
12	Guyana	11.6 (9.4–14.2)	12	Guyana	11.5 (9.5–14.0)
13	Greece	11.2 (10.−11.9)	13	Bahrain	11.3 (10.9–11.8)
14	Nepal	11.1 (7.6–16.1)	14	Nepal	11.2 (7.6–16.2)
15	Türkiye	11.0 (9.4–13.0)	15	Brazil	11.1 (10.7–11.6)
16	Botswana	10.8 (4.5–24.8)	16	Côte d’Ivoire	11.0 (10.0–12.1)
17	Guatemala	10.5 (5.5–19.1)	17	Türkiye	11.0 (10.0–12.1)
			18	Botswana	10.8 (4.5–24.8)
			19	Uganda	10.0 (6.1–16.0)
			20	United States of America	10.0 (9.6–10.4)

17 countries had a preterm rate 10% or higher in 2010, and 20 countries in 2020

^a^Credible interval

Almost half (45%) of all preterm births in 2020 occurred in just five countries: India, Pakistan, Nigeria, China and Ethiopia (Fig. 3) despite accounting for a lower proportion of the world’s live births. India had the highest number of preterm births in 2020 (3.02 million, 95% CrI: [2.2, 4.0 million]), accounting for more than 23% of all preterm births worldwide) with Pakistan, Nigeria and China each having more than three quarters of a million preterm babies in 2020. Countries with large numbers of preterm births reflect both larger numbers of total births, and higher preterm birth rates [12].

**Fig. 4:**
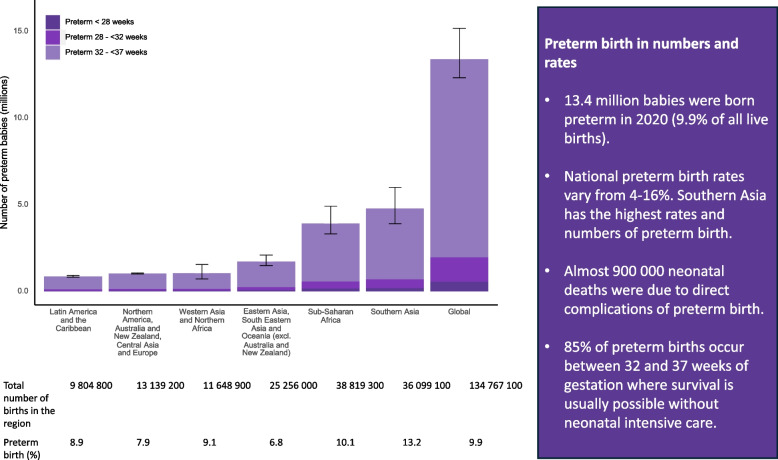
Preterm birth by gestational age and region in 2020 Data Source: Adapted from WHO/UNICEF preterm estimates. Ohuma, Moller, Bradley et al. [12]

At the regional level, the largest burden of preterm birth remains in Southern Asia, where 4.8 million babies (95% CrI: [3.9, 6.0 million]) were born preterm in 2020, including more than 700 000 at <32 weeks, with the highest risks of mortality and long-term consequences (Fig. 4). Sub-Saharan Africa accounted for 3.9 million (95% CrI: [3.3, 4.9 million]) preterm births, almost 600 000 of them at <32 weeks [12].

In 2020, nearly 1.2 million preterm newborns are estimated to have been born in the 10 most fragile countries affected by humanitarian crises (Afghanistan, Chad, Central African Republic, Democratic Republic of the Congo, Myanmar, Somalia, South Sudan, Sudan, Syrian Arab Republic and Yemen) [18]. Many women and preterm babies in these settings face increased challenges in accessing care, especially higher-level care [19] The complete country, regional and global estimates can be found in Additional file 1.

### Deaths and lifelong impacts for survivors of preterm birth

Neonatal conditions remain the fifth leading cause of death globally (after ischaemic heart disease, stroke, chronic obstructive pulmonary disease and lower respiratory tract infections) and the leading cause of loss of human capital (e.g. Disability-adjusted life years (DALYS)) [20].

This high burden of DALYs from neonatal conditions is due primarily to deaths at an early age. Preterm birth complications are the leading cause of mortality among children under five years of age, as well as a leading risk factor for other causes of death such as infections [21]. Almost a million children (0.9 million) were estimated to have died due to direct complications of preterm birth in 2022, and over a third of all neonatal deaths worldwide are estimated to be due to direct complications of preterm birth [21].

**Fig. 5:**
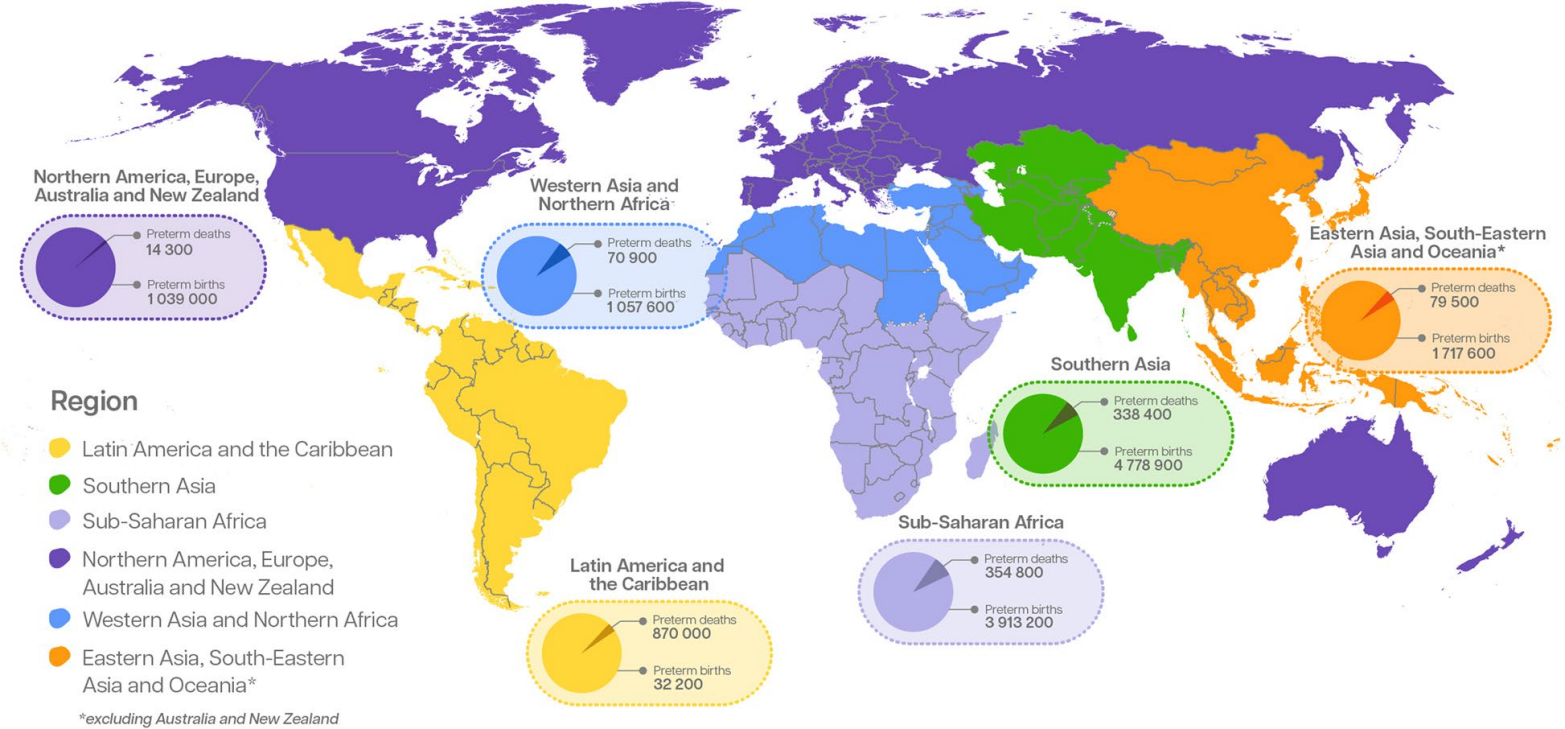
Regional variation in the proportion of preterm births resulting in neonatal deaths in 2020 In each pie chart darker shading in indicates proportion of preterm births with neonatal death (first 28 days), by region. Data sources: Preterm birth numbers by country from Ohuma et al. [12]. Mortality estimates generated by applying country specific 2019 preterm cause-specific neonatal death proportions from Perrin et al. [22] to 2020 country-specific live birth estimates from World Population Prospects (https://population.un.org/wpp/) [23]

Inequalities in care between and within countries result in unacceptably large survival gaps for babies born preterm. While higher-resourced settings have near-universal survival for those born over 28 weeks’ gestation, mortality rates remain high in areas where access to care is limited, even for babies born up to 32 weeks’ gestation. Resulting in substantial regional variations in preterm survival (Fig. 5). These numbers reflect continued stagnation in preterm birth rates and missed opportunities to improve care for preterm babies [19, 21].

Preterm birth is also associated with long-term health consequences in survivors, including respiratory and cardiovascular complications and neurodevelopmental impairments. These impairments can vary from major disabilities, such as diplegia, particularly for those born the most preterm, to less severe outcomes. Importantly, new research shows that being born even a few weeks preterm can increase the risk of learning and behavioural spectrum disorders. Since most preterm births (85%) occur between 32 and 37 weeks, a significant portion of preterm babies are at risk, contrary to previous assumptions that only extremely preterm born babies were vulnerable. Importantly, even those born at between 37 and less than 40 weeks have a slightly elevated risk of death and an elevated risk of adverse neurodevelopmental outcomes, notably behavioural conditions compared to babies born at term [22, 24, 25].

Disability-free survival is a sensitive marker of the quality of care provided to preterm babies. Many impairments associated with preterm birth, including blindness and visual impairment due to retinopathy of prematurity, are preventable. Recent decades have witnessed an increase in retinopathy of prematurity cases, particularly in East and South Asia, even among moderately preterm infants [26, 27]. Improving safe oxygen use and expanding screening and treatment programmes in parallel with increased access to inpatient neonatal care is essential to avoid a repeat of the epidemics of blindness previously observed in Western Europe, the United States, and Latin America [26].

These outcomes impact not only the individuals born too soon, but also their families, caregivers, communities, healthcare systems and wider society [28].

## Programmatic priorities

This global burden of preterm birth remains a top issue with limited progress in primary prevention. Affecting all countries, preterm birth should be prioritised on the global health agenda [29]. Evidence shows that this burden can be reduced [30]. If countries are to achieve SDG target 3.2 to end preventable neonatal deaths, and also transform human capital through the whole life-course, urgent action is needed both to prevent preterm birth and to improve the quality of care for those born preterm. Although stillbirths are often left out of this equation, most stillbirths are born preterm, and greater investment in primary prevention of preterm births could help to reduce the 1.9 million stillbirths every year [13, 31].

Here we summarise the measurement priorities for two tracks for addressing the burden of preterm birth, in terms of prevention and care [29].

### Track 1 measurement priorities: preventing preterm birth by using and improving data

Despite little progress in reducing preterm births at global and regional levels, progress has been seen in a few, predominantly higher-income, countries. Reducing preterm births in all settings will require addressing the drivers of preterm birth, which may be context- specific. It is therefore essential that strategies to reduce preterm birth rates are setting-specific, and approaches are generated from low- and middle-income countries (LMICs) as well as high-income countries [32].

Table 2 provides an overview of known risk factors in both spontaneous and provider-initiated preterm births. In settings with high caesarean section rates it is crucial to consider the incentives for this and to increase knowledge for all concerned regarding risks to women and their babies due to non-medically indicated caesarean section [30, 33]. For spontaneous preterm birth, many of these interventions can be delivered through high-quality antenatal care. See accompanying paper in this series for details of some of these programmatic approaches [34]. However, intersectoral interventions targeting whole populations, such as reducing smoking and obesity and improving air quality, are also important for reducing preterm birth overall [35].

**Table 2:** Preterm birth risk factors and prevention strategies

**Type**	**Risk factors**	**Examples**	**Examples of prevention strategies **^**a**^
Spontaneous preterm birth^b^	Age at pregnancy and pregnancy spacing	Adolescent pregnancy, advanced maternal age, short inter-pregnancy interval	Preconception care, including access to family planning from adolescence, after birth and throughout reproductive years
	Multiple pregnancies	Increased rates of twin and higher-order pregnancies with assisted reproduction	Introduce and monitor policies for best practice in assisted reproduction
	Infection	Urinary tract infections, asymptomatic bacteriuria, malaria, HIV, syphilis, chorioamnionitis, and bacterial vaginosis	Sexual health programmes aimed at prevention and treatment of infections prior to and during pregnancy Intermittent preventive treatment of malaria (context-specific), antenatal screening for lower genital tract infections and asymptomatic bacteriuria
	Underlying chronic medical conditions	Diabetes, hypertension, anaemia, asthma, thyroid disease, and HIV	Maximize preconception control for pre-existing conditions, as well as screening and prompt management during pregnancy
	Nutritional	Undernutrition and micronutrient deficiencies	Assess and treat low nutritional status prior to conception and in early pregnancy. Consider supplementation (e.g. iron folate and zinc supplementation) for pregnant women without systemic illness
	Lifestyle and work-related	Smoking, excess alcohol consumption, recreational drug use, excess physical work and activity	Adopt laws and rights-based approaches to protect pregnant women, and ensure maternity leave. Behavioural and community public health interventions targeting pregnant women and women of reproductive age, e.g. pharmacological interventions for smoking cessation
	Environmental	Exposure to indoor and ambient air pollution, and heat stress	Public health measures, antenatal counselling, avoidance of air pollution and excessive heat where possible
	Maternal psychological health	Depression and violence against women	Antenatal screening where capacity to provide a supportive response is available
	Genetic and other	Genetic risk (e.g. family history), cervical incompetence, intrauterine growth restriction, and congenital abnormality	Individual-specific interventions, e.g. cervical cerclage for women with singleton pregnancy and high risk of preterm birth
Provider-initiated preterm birth	Induction or caesarean birth for maternal indication	Common indications include: pre-eclampsia/eclampsia, placental abnormalities (e.g. placenta accrete) and preexisting maternal conditions	Not applicable
	Induction or caesarean birth for fetal indication	Common indications include severe fetal growth restriction	Not applicable
	Induction or caesarean birth without medical indication	Non-medically indicated, due to physician or patient preferences or incentives	Programmes and policies to reduce the practice of non-medically initiated preterm birth. Midwifery-led continuity models of care have proved effective

^a^Updated from Blencowe et al. [33] and Medley et al. [30]

^b^Includes preterm birth following preterm pre-labour rupture of membranes

### Track 2 measurement priorities: caring for preterm babies across the life-course by using and improving routine data

**Fig. 6:**
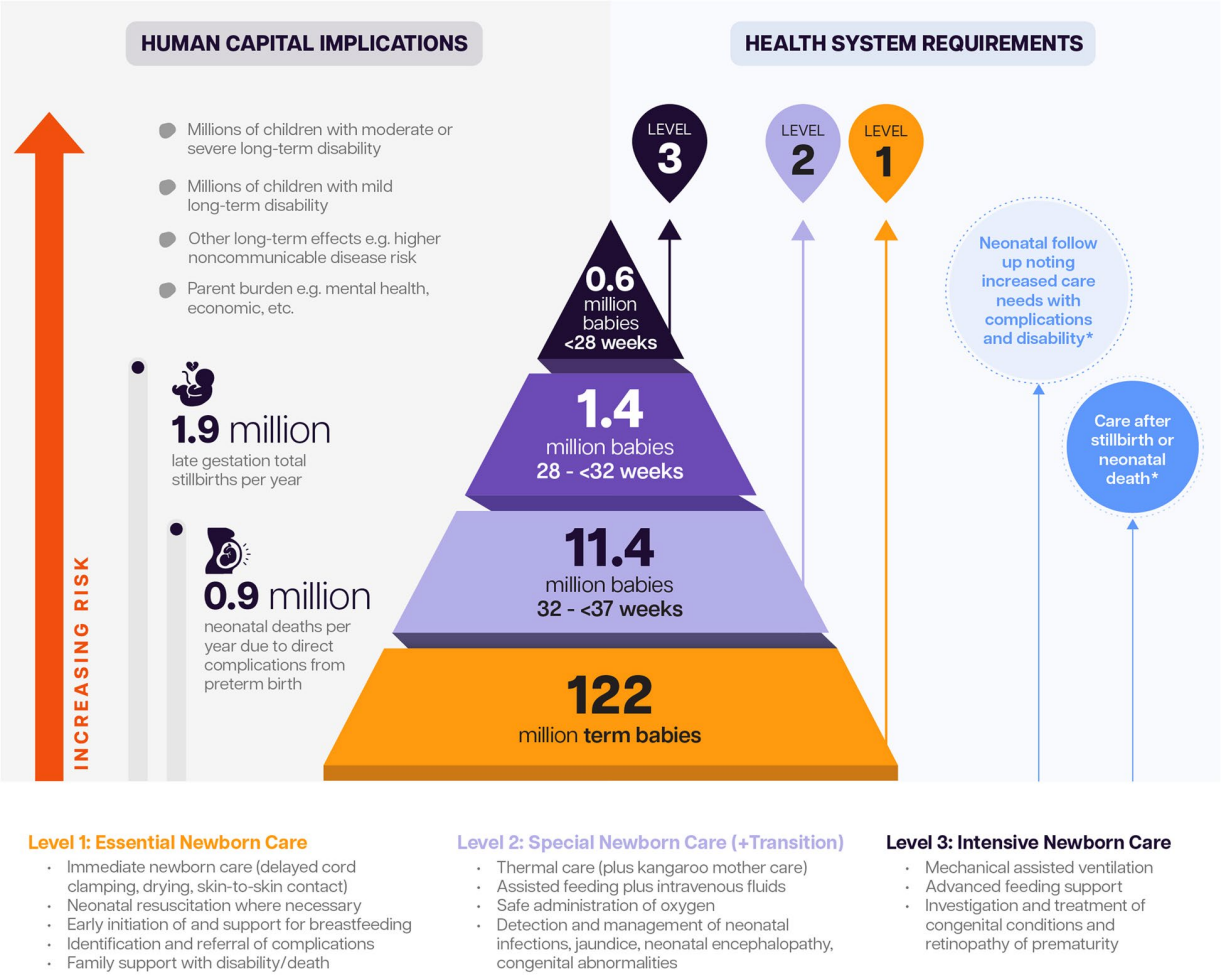
13.4 million preterm births in 2020: human capital implications and health system requirements. Around three quarters of stillbirths are preterm in high- and upper-middle-income settings [13] ^e ^In addition to newborn care, health systems need to be designed to provide care after a stillbirth or death in this high-risk population and to cater throughout the care continuum for the increased health-care needs associated with preterm birth [19]. Adapted from Lawn et al. [13]

Some countries have made notable progress over the past decade in improving care for small and sick newborns [19]. Accurate data on gestational age allow preterm babies to be categorised into subgroups, which is essential to enable countries to assess and plan for the level of care needed, since gestational age is a predictor of the risks of mortality and long-term disability. All babies, regardless of gestational age, require essential newborn care, and most babies born preterm require additional support or special newborn care, with a smaller number, including those born at <28 weeks, requiring intensive care (Fig. 6) [13].

Many settings lack systems for routine follow-up of at-risk newborns, have gaps in care, and lack adequate information about the short-, medium- and long-term impacts of preterm birth on human capital. An accompanying paper in this series provides more details on health- care services for preterm newborns, integrated within care for all small and sick newborns, as well as at-risk neonatal follow-up [19].

Encouragingly, some countries are improving data on preterm birth, incorporating these data in national systems and using this information to track progress and inform programmes. For example, countries are increasingly using web-based national data platforms, which have facilitated increased reporting of national data on preterm and low birthweight births (LBW) (e.g. 80 low and middle-income countries use DHIS-2) [36, 37]. Further investments are needed to improve the representativeness and quality of the data produced [38].

## Pivots

Data are needed to track progress and to inform action. Gaps in routine data availability, quality and reporting in many LMICs currently limit the evidence available on preterm births [36]. This limitation can be overcome by improving data collection at the individual level for every baby, strengthening data systems to capture this information and ensuring that the data are made available.

### Pivot 1: count every baby, fully including stillbirths, improve gestational age and birth weight measurement

**Fig. 7:**
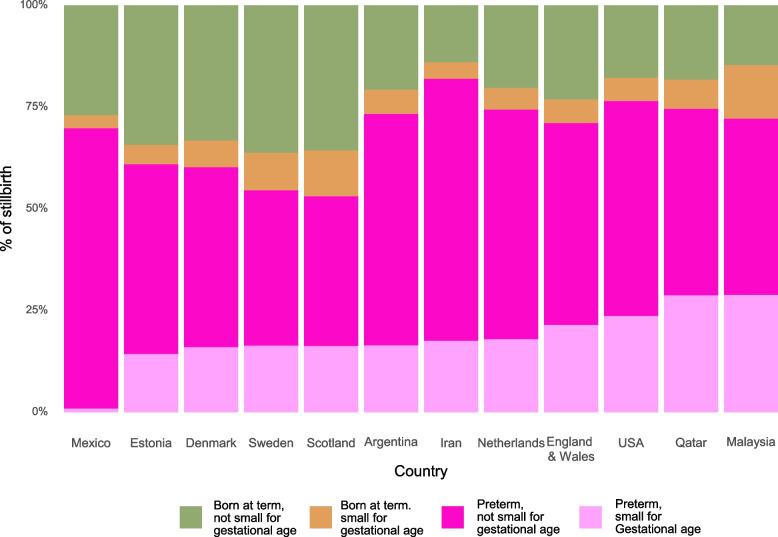
Four types of vulnerable newborns amongst stillbirths Source: Lawn et al. [13]

Improving data on preterm birth requires counting every baby everywhere, whether live or stillborn, and recording their gestational age and birth weight. Preterm birth data currently primarily focus on live births, overlooking a major part of the burden. A substantial proportion (74.3%) of the estimated 1.9 million late-gestation stillbirths (28 or more weeks) worldwide occur before 37 weeks’ gestation [39]. Stillbirth and preterm birth are associated with similar vulnerability pathways, whereby preterm labour can result in stillbirth and, conversely, in-utero fetal death may result in preterm labour [31]. Analyses of 12 upper-middle- and high-income countries (0.6 million stillbirths ≥ 22 weeks’ gestation) showed that around 74.3% of stillbirths in these settings were preterm (Fig. 7) [13, 39]. Further analyses showed that small for gestational age was strongly associated with stillbirth risk, and the highest risk ratios for stillbirth was in gestations <37 weeks across all resource settings [40–45].

To track and change progress for the world’s most vulnerable babies it is essential to include and count stillbirths alongside live-born preterm babies and the 0.9 million associated neonatal deaths [21, 39]. Omitting those born still distorts the quantification of the total burden of those “born too soon”, especially in comparisons between different settings and when assessing trends over time. For example, with better obstetric monitoring, more deliveries to prevent stillbirth could increase the rates of preterm births among live births. Interventions to address preterm birth (e.g. due to infections or pre-eclampsia) may also reduce stillbirths, but the full impact of such interventions will only be measurable by recording stillbirths [46].

The WHO minimum perinatal dataset for every baby includes gestational age, sex and birth weight, as well as mode of delivery including caesarean section. It is essential that these data are collected for every birth, and are of good quality, in order to inform future action to prevent preterm births [13, 47, 48]. To further improve data on causes and prevention, it is also important to measure the number of children per birth so that multiple births can be accounted for.

Gaps in gestational age measurement are commonly cited as barriers for preterm birth data. However, substantial advances over the last decade make the availability of accurate gestational age measurement possible in most countries.

Antenatal care coverage has increased, and ultrasound is more often available and at lower cost. While first trimester pregnancy ultrasound is still considered the gold standard for pregnancy dating, there is now evidence that sonography up to 22–24 weeks provides acceptable accuracy, which is important given that the majority of women worldwide start antenatal care after the first trimester [49, 50].

Recent ultrasound innovations may also increase the accuracy of gestational age assessment for pregnancies >24 weeks [4–7]. In view of these advances, all countries should strive to invest in improving the accuracy of gestational age determination in the first trimester, use these data for better individual care and collate them in routine data systems.

Birth weight is a long-standing measure for assessing babies. Although data on weight have been routinely collected for decades, the widespread introduction of digital scales over the last decade enables greater accuracy [51].

Routine standardised assessment of size for gestational age is also now possible, given the improvements in measurement of gestational age and birth weight over the past decade, coupled with the release of the INTERGROWTH-21^st^ growth standards [52]. Pivoting to including size-for- gestational-age assessment for every baby will facilitate more granular classification of risks for vulnerable newborns, for example by newborn type, combining size-for-gestational-age with gestational age. This information will improve individual clinical care, enable better assessment of long-term outcomes and help drive accountability for progress towards multiple SDGs, as well as global nutrition targets since preterm birth is a major driver of LBW [13].

### Pivot 2: strengthen routine data systems for tracking vulnerable babies, including follow-up care

**Fig. 8:**
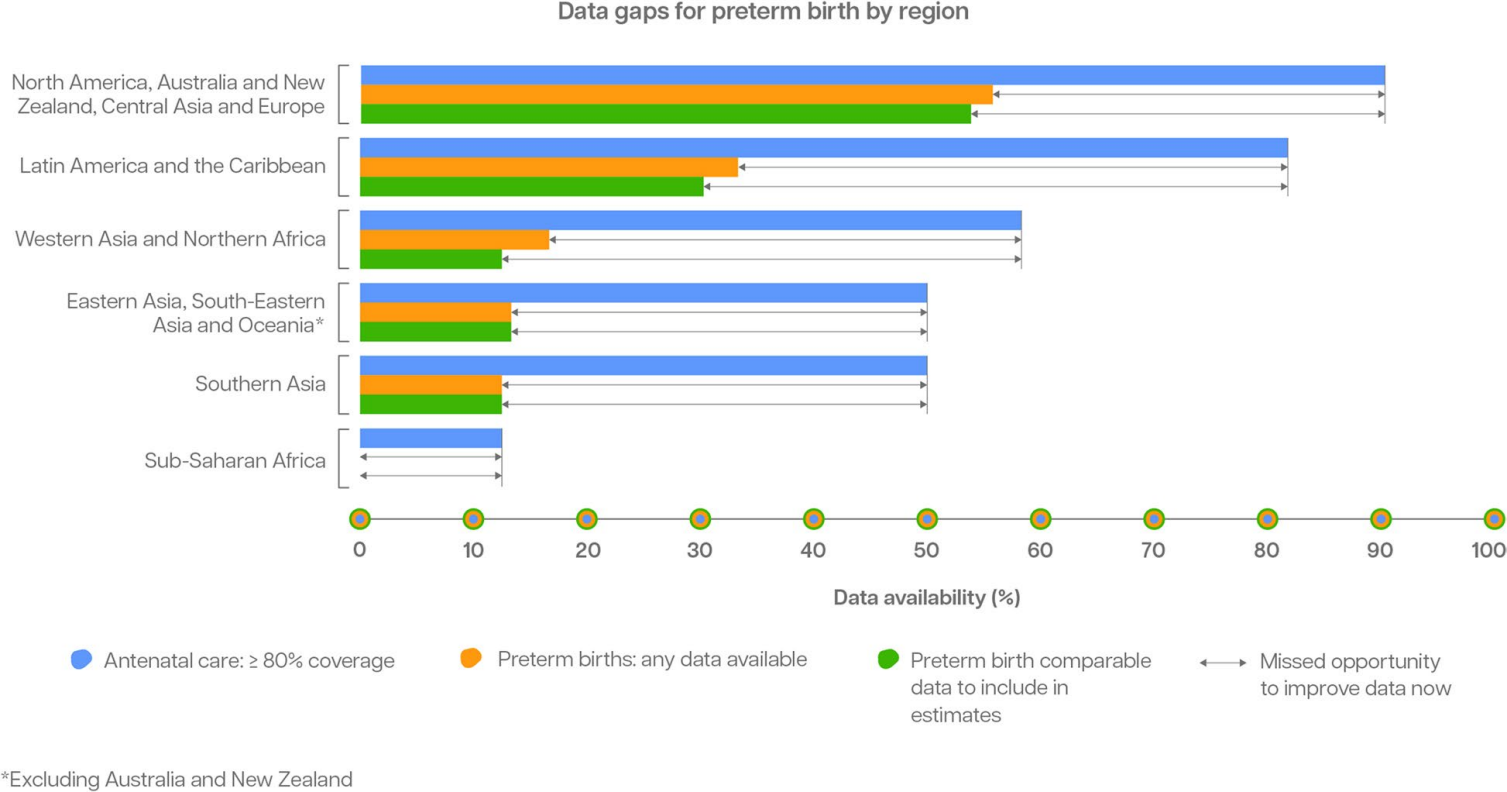
Missed opportunities to improve capture of national administrative data on preterm birth, by SDG region, 2010–2020 ^1^Excluding Australia and New Zealand. Figure represents gaps for 194 WHO Member States and the occupied Palestinian territory, including east Jerusalem. ≥80% coverage of four antenatal visits was used as a proxy for early antenatal attendance and gestational assessment, noting that this is an imperfect proxy. Adapted from Lawn et al. [13]

Despite the increasing rates of births in health facilities over the past decade (now around 80% worldwide) and investments in health information systems, opportunities have been missed to improve preterm birth data. Recording gestational age information in labour wards for all facility births, and collating aggregate data through health information systems, would enable the tracking of progress at national and subnational levels [13, 53]. Only 33% of countries (64 out of 195) routinely collected national preterm birth data of sufficient quality to be included in the latest round of global, regional and national preterm birth estimates for 2010-2020 (64 of 103 (62%) of the preterm database) [12]. However, 81% (158) have useable LBW data, partly due to this being measured in population-based surveys [54]. Gaps in routinely collected data on preterm birth are most marked in Southern Asia, South-Eastern Asia and sub-Saharan Africa which, on average are the regions with the highest rates and absolute numbers of preterm births (Fig. 8) [12].

Reliable preterm birth data are achievable in countries that have both high rates of births in health facilities and robust health information data systems. Improving routine data systems in these countries requires attention and adequate investment. In countries with weak routine data systems and fewer births in health facilities, and in settings affected by conflict or other humanitarian crises, innovative strategies are needed to collect and use data on preterm birth.

While the first step towards overcoming data gaps for preterm birth is to ensure that every baby, live- or stillborn, is counted and recorded in relevant data systems, it is also necessary to improve data quality to maximize comparability. Over the last decade, tools have been developed to assess the quality of data on newborns within routine health information systems [55, 56].

Most routine health information systems record only aggregate data in their electronic data systems. Over the next decade, increasing national coverage of electronic-based individual-level data will be an important step. Individual-level data are crucial for individualized care and quality improvement on maternity wards and newborn care wards. When setting up such a system it is important to start small and focus on high capture and data quality, expanding the variables later. Having a standard dataset, for example for neonatal inpatient care, can address programmatic quality issues, such as hypothermia at admission for inborn and outborn babies, and by weight group. In settings with more robust data collection systems, individual-level data with unique identifiers can enable linkages to track short- and long-term outcomes following preterm birth and other vulnerable newborn types across populations, in order to reduce gaps in follow-up care [13]. Such electronic cohorts also enable long-term human capital outcomes to be tracked across the life- course, including health, education, welfare and economic outcomes [57]. Currently there are very few such studies in LMICs.

Individual-level data facilitate equity analyses. Disaggregated data can be used to identify the geographical and other population groups with the worst outcomes, enabling resources to be allocated to the populations in greatest need.

Improved data are also needed to understand the drivers of preterm birth in different settings. A first step towards this is to collect information for every baby on whether birth was spontaneous or provider-initiated, using more standard definitions and applying this data to inform interventions, such as reducing non-medically indicated caesarean sections [58].

### Pivot 3: improve use of data for action and accountability

The final pivot for data on preterm birth and other small babies is to ensure that these data are used to drive accountability for action. This requires data, not only on preterm birth rates and outcomes, but also on what works and how to address key risk factors and improve coverage of high-quality care for preterm babies at all levels of the health system. Data need to be accessible to a range of stakeholders, including governments and ministries of health, and public health policy planners and implementers. Importantly, they also need to enable families and civil society to engage actively in accountability processes. A range of formats is likely to be required, such as annual health reports, maternity reports, dashboards, open databases and lay summaries. One example of this is the March of Dimes’ annual report of state-level preterm birth rates across the United States of America, which includes: the annual rate of preterm births, a target for the reduction of this rate, mortality rates, and disaggregation of these statistics by factors such as race [59].

## Conclusion

Preventing preterm birth is a fundamental step for achieving SDG target 3.2 to end preventable child and neonatal deaths, as well as many other targets related to economic growth and human capital. There has been no measurable change in preterm rates the past decade in any regions, and the global burden remains high with an estimated 13.4 million newborns born preterm in 2020. Programmatic priorities need to be tailored to context, but there are approaches that can be universally applied such as immediate opportunities to improve care of affected babies. More innovation is needed for methods of preterm birth prevention and the implementation of the known approaches. Increasing data on gestational age and birthweight in routine systems is doable and necessary to enable individual care and national level progress for preterm babies, and crucially including stillbirths in these data is essential for the whole burden of preterm births to be quantified. A decade ago, our first preterm estimates in the original “Born Too Soon: The Global Action Report on Preterm Birth” report led to more visibility for preterm birth worldwide – now is the time to use the data we have to speed up real change in every country.

## Supplementary Information

Additional file 1. National, Regional and Global estimates of Preterm birth in 2020, with trends form 2010 [12].

## Data Availability

All data are available in the paper or in supplementary files. Additional information is available at www.borntoosoonaction.org and https://github.com/WorldHealthOrganization/mca-pretermbirths-analysis/tree/main.

## Abbreviations

SDG: Sustainable Development Goals
WHO: World Health Organisation
UNICEF: United Nations Children’s Fund
DALYS: Disability-adjusted life years
LMIC: Low- and Middle-Income Country
LBW: Low birth weight
CrI: Credible interval
CI: Confidence interval
